# Musical and electrical stimulation as intervention in disorder of consciousness (DOC) patients: A randomised cross-over trial

**DOI:** 10.1371/journal.pone.0304642

**Published:** 2024-05-31

**Authors:** Simona Spaccavento, Giulio Carraturo, Elvira Brattico, Benedetta Matarrelli, Davide Rivolta, Fabiana Montenegro, Emilia Picciola, Niels Trusbak Haumann, Kira Vibe Jespersen, Peter Vuust, Ernesto Losavio

**Affiliations:** 1 Istituti Clinici Scientifici Maugeri IRCCS, Institute of Bari, Bari, Italy; 2 Department of Education, Psychology, Communication, University of Bari Aldo Moro, Bari, Italy; 3 Center for Music in the Brain, Department of Clinical Medicine, Aarhus University & Royal Academy of Aarhus/Aalborg, Aarhus, Denmark; La Sapienza University of Rome, ITALY

## Abstract

**Background:**

Disorders of consciousness (DOC), i.e., unresponsive wakefulness syndrome (UWS) or vegetative state (VS) and minimally conscious state (MCS), are conditions that can arise from severe brain injury, inducing widespread functional changes. Given the damaging implications resulting from these conditions, there is an increasing need for rehabilitation treatments aimed at enhancing the level of consciousness, the quality of life, and creating new recovery perspectives for the patients. Music may represent an additional rehabilitative tool in contexts where cognition and language are severely compromised, such as among DOC patients. A further type of rehabilitation strategies for DOC patients consists of Non-Invasive Brain Stimulation techniques (NIBS), including transcranial electrical stimulation (tES), affecting neural excitability and promoting brain plasticity.

**Objective:**

We here propose a novel rehabilitation protocol for DOC patients that combines music-based intervention and NIBS in neurological patients. The main objectives are (i) to assess the residual neuroplastic processes in DOC patients exposed to music, (ii) to determine the putative neural modulation and the clinical outcome in DOC patients of non-pharmacological strategies, i.e., tES(control condition), and music stimulation, and (iii) to evaluate the putative positive impact of this intervention on caregiver’s burden and psychological distress.

**Methods:**

This is a randomised cross-over trial in which a total of 30 participants will be randomly allocated to one of three different combinations of conditions: (i) Music only, (ii) tES only (control condition), (iii) Music + tES. The music intervention will consist of listening to an individually tailored playlist including familiar and self-relevant music together with fixed songs; concerning NIBS, tES will be applied for 20 minutes every day, 5 times a week, for two weeks. After these stimulations two weeks of placebo treatments will follow, with sham stimulation combined with noise for other two weeks. The primary outcomes will be clinical, i.e., based on the differences in the scores obtained on the neuropsychological tests, such as Coma Recovery Scale-Revised, and neurophysiological measures as EEG, collected pre-intervention, post-intervention and post-placebo.

**Discussion:**

This study proposes a novel rehabilitation protocol for patients with DOC including a combined intervention of music and NIBS. Considering the need for rigorous longitudinal randomised controlled trials for people with severe brain injury disease, the results of this study will be highly informative for highlighting and implementing the putative beneficial role of music and NIBS in rehabilitation treatments.

**Trial registration:**

ClinicalTrials.gov identifier: NCT05706831, registered on January 30, 2023.

## Introduction

Music is a powerful stimulus affecting human perceptual, cognitive and motor brain functions, including movement, language, memory, attention, and emotions [1]. Musical activities in a clinical setting are highly motivational, as they activate the dopaminergic mesocorticolimbic reward circuitry, allowing for their reiteration over time, which leads to learning and ultimately to brain plasticity [2]. Indeed, a recent meta-analysis of 84 neuroimaging studies using structural magnetic resonance imaging (MRI) and functional MRI (fMRI) evidenced plastic changes in the physiology and anatomy of primary and non-primary sensory areas of the supratemporal lobe as well as cortical and subcortical motor circuits in frontostriatal regions as a consequence of years of musical practice in healthy musicians [3].

The potential role of music for the neurorehabilitation in the realm of severe acquired brain injury (ABI), such as disorders of consciousness (DOC), is still largely unexplored. ABI includes a wide range of conditions caused by traumatic events (e.g., traumatic brain injury), by vascular event (e.g., ischemic or haemorrhagic stroke), or by cerebral anoxia, toxic or metabolic insults (see [4] for a review). These neurological conditions can result in mild to severe impairments of motor and cognitive functions as well as in emotional and neuropsychiatric symptoms, which in turn may lead to a significant level of disabilities and poor functional outcome. Various rehabilitation techniques and treatments have been developed for the neuropsychological deficits associated with ABI (e.g., aphasia, neglect, attention and memory deficits, etc.), ranging from behavioural to cognitive interventions [5]. However, current evidence on rehabilitation techniques of ABI highlights relevant methodological and measurement inconsistencies, mainly due to the heterogeneity of this group of patients. Furthermore, rehabilitation interventions are often very complex and target multiple outcomes, which can be difficult to evaluate independently.

In fact, ABI could result in DOC, which, in turn, based on the observable behavioural features and their inferred relationship to level of consciousness, is clinically manifested as coma, vegetative state (VS), or minimally conscious state (MCS) [6]. Coma is a condition clinically defined as the complete loss of spontaneous or stimulus-induced arousal [7]. This condition is a self-limiting state that typically resolves within 2 weeks and transitions into either VS or MCS [6]. VS is a condition of wakeful unconsciousness, which is diagnosed when spontaneous eye-opening remerges, although the absence of any discernible evidence of language comprehension, verbal or gestural communication, or reproducible purposeful behavioural responses to visual, auditory, tactile or noxious stimuli persist [8]. Lastly, MCS is a condition of severely altered consciousness characterised by minimal yet definite behavioural evidence of self or environmental awareness. It is a condition that usually results from the improvement in consciousness after coma or VS [6].

For the diagnosis of DOC type, the neurologist is typically aided by electroencephalography (EEG), a widely available routinely-used neurophysiological technique that records the synchronised post-synaptic electrical activity of neurons. In DOC patients, EEG signal recorded during rest is clearly atypical; it is indeed characterised by a generalised slowing in the theta or delta ranges and it presents other patterns including epileptiform activity, burst-suppression and alpha-coma. EEG is highly functional for the differentiation of DOC types: for patients without preserved consciousness, EEG shows predominant low, mostly delta, frequency, combined with a marginalization of the higher frequencies, both in terms of the global power of brain activity and in functional connectivity patterns; for MCS patients the opposite EEG pattern is seen: higher frequency bands are preserved both in global power and in functional long-distance connections [9].

Additional to the analysis of oscillatory patterns in the resting-state EEG, also the evoked responses to passive stimulation, typically auditory, have been studied in DOC patients. Among these responses, the one that has biomarker features, meaning it also has some predictive power in relation to the awakening outcome of the patient, is N100. According to a predominant theory of brain function (the Predictive Coding Theory or PCT; [10]), the N100 tackles the predictive process of anticipating the sensory environment based on learning its properties by extracting the events’ statistical probabilities and continuously updating its internal model. In the auditory system, a decreased N1 reveals a memory process which is further modulated by the precision of the context: if a context is uncertain, such as when a listener has never heard a song before, the N1s to all sounds of that song are enhanced as compared to when the tones of the song are learned [10].

Given the clinical and neurophysiological features of DOC patients, music may represent a useful means that could function as an alternative communication channel for multiple reasons. Music represents a nonverbal auditory stimulus with a marked emotional salience (e.g., [11, 12]); as such, it may be regarded as an optimal stimulus in contexts where cognition is severely compromised and stimuli with personal meaning may produce relevant behavioural changes [13–15]. This might be particularly relevant as the most successful rehabilitation interventions are those that are tailored to the individual [16]. Moreover, music-based interventions are based on integrity of the hearing system, which is usually intact in DOC conditions, despite a frequently impaired visual system [17]. Second, music listening may be seen as a kind of enriched sensory stimulation and, hence, a subtype of cognitive rehabilitation strategies; these strategies are based on the idea that an enriched environment benefits brain plasticity and improves recovery of injured brains [18]. Some studies [19, 20] showed as music affect neural networks, accelerating brain plasticity and avoiding sensory deprivation. Multisensory stimulation treatment, based on auditory, visual, tactile, olfactory and gustatory stimuli, can improve the level of consciousness, above all when the stimuli are of high emotional relevance. This aspect is very important also for music stimulation that ought to be used as rehabilitation treatment in DOC.

Neuropsychological studies have shown that music increases arousal and attentions in DOC patients, with significant cortical and Autonomic Nervous System (ANS) activity [21]. Specifically, music accelerates brain plasticity by increasing the activity in frontal, temporal, parietal and subcortical processes, with an important effect on the recovery process [22]. Moreover, in DOC patients, the emotional content of salient music leads to an activation of limbic structures [23, 24], as well as an increase in eye blinks during the exposure to preferred songs [21]. The impact of music on DOC patients is also visible in their EEG responses. O’Kelly et al. [21] investigated the cognitive correlates of music perception in DOC patients and found that passive exposure to preferred songs increased EEG amplitude in frontal midline theta and alpha in both VS and MCS cohorts. These findings resonate with what has been observed even in healthy subjects: the spectral power of the frontal midline theta is increased during listening to preferred pleasant music in contrast to unpleasant music [25]. The salience of reward circuits in DOC is confirmed by the fact that this condition is often associated with widespread impairment of the dopaminergic system, as well as a reduced level of dopamine in the cerebrospinal fluid [26]. Therefore, interventions aimed at stimulating dopamine release may be particularly appropriate for DOC. In fact, a context of preferred music is deemed to be more appropriate than a neutral context to the expression of residual cognitive functioning in patients with DOC [27]. In sum, music-based interventions have consistently been associated with behavioural and neural improvements among DOC patients (for a review, see [28]).

A further group of rehabilitation strategies for brain-lesioned patients, including DOC patients, consists of neuromodulation aimed to alter the physiology of the neural circuits responsible for the behaviour under investigation. Among several neuromodulation approaches, there are pharmacological interventions (e.g., amantadine hydrochloride) [29], deep brain stimulation (for a review see [30]), and Non-Invasive Brain Stimulation techniques (NIBS) [31]. NIBS exists in various forms, although one of the most adopted in clinical settings is transcranial electrical stimulation (tES). This technique consists of delivering a small (1–2 mA) current via two electrodes placed on the scalp. According to the stimulation parameters, tES affects neural excitability and has plastic effects (mediated by NMDA-receptors) [32]. These currents, cathodal (-) or anodal (+), generate an electric field that can modulate spontaneous neural activity by interfering with the membrane potential of the underlying neuronal compartments by inducing, respectively, hyperpolarization (inhibiting effect) or depolarization (facilitating effect) [33]. Importantly, the induced changes become more stable and long-lasting (i.e., aftereffects) when stimulation is repeated several times [34]. Key targets for stimulation in the neurocognitive domain are the dorsolateral prefrontal cortex (DLPFC), especially in working memory, attention, and executive functions, and the posterior parietal cortex (PPC), which plays a critical role in visual spatial perception and attentional processes [35]. Several tES studies show improvements in clinical (e.g., GCS, CRS-R, cognitive, motor) and surrogate (i.e., EEG, TMS, fMRI) outcomes of patients with DOC [36–38], for a review see Kuo et al. [39], although this effect is more evident among MCS patients rather than among unresponsive wakefulness syndrome patients (VS/UWS) [40]. NIBS is, thus, considered a useful tool in the rehabilitation of clinical populations such as patients with DOC [41]; for instance, tES has been used as a potential adjuvant treatment to improve symptoms in patients with hemispatial neglect after stroke [42]. However, there is an increasing understanding that patients do not homogeneously respond to NIBS]. This might be ascribed to the fact that the methodology adopted in tES studies is characterised by a large variability as concerns stimulation parameters, participant characteristics as well as the characteristics of associated rehabilitation treatment.

With this study we propose a novel rehabilitation protocol that combines music-based intervention and NIBS in neurological patients aimed to (i) assess the residual neuroplastic processes existing in DOC state, and (ii) evaluate the putative modulation of these processes by a non-pharmacological 2-weeks intervention combining electric (tES) and auditory (music) stimulation. The musical stimulation will consist of musical pieces that vary in familiarity and complexity (from pieces chosen from the favourite ones for the patient to novel pieces following classical tonal style up to atonal pieces). We hypothesize that tES, altering the physiology of neural circuits, can increase the plastic effect of music stimulation and thus increase the responsiveness of the patient. As far as we know, there are no studies in the literature that have evaluated the effectiveness of the association of these treatments in DOC patients Furthermore, an additional novel aspect will be the use of autobiographical music, hence emotionally relevant to the patients.

In addition, we also want to ascertain the influence of this rehabilitation protocol on caregiver’s burden and psychological distress, since acquired brain injury has an important impact on physical, psychological, social and also financial conditions of caregivers; their entire life is modified in order to take care of the patient, causing a strong emotional burden, with high levels of psychophysiological distress, depression, anxiety and a more complex symptomatology called prolonged grief disorders [43, 44]. In this regard, an aim of this study is to evaluate the caregiver’s psychological burden both before and post rehabilitation treatment in order to assess the effect of music stimulation and brain stimulation also on caregiver’s emotional condition.

The outcome measures will be clinical, namely based on the neurologist’s observations of clinical improvement and responsiveness, measured through specific scales as well as neurophysiologically. For the latter, continuous EEG is expected to display a decrease of power spectra of slow frequency bands (delta-theta) and an increase in power for faster frequency bands (alpha, beta, gamma). We also expect to evaluate residual memory and learning processes in DOC patients by measuring a higher EEG N100 component amplitude after compared to before treatment. Also, amplitudes of the later-stage auditory P2 and latencies of the N100 and P2 will be explored. More broadly, the present intervention combining music exposure with electric neuromodulation is expected to produce increased evoked responses indexed in EEG measures.

## Material and methods

### Participants

All patients consecutively admitted to the Neurorehabilitation Unit at the ICS Maugeri (Bari, Italy) with Vegetative State (VS) or minimally conscious state (MCS) [45] will be assessed. Our hospital is a reference centre for patients with severe acquired brain injury; a letter will be sent to the acute hospitals from which patients come with the aim of informing them of the research project. Inclusion criteria will be the following: clinical diagnosis of VS or MCS according to standardised clinical diagnostic criteria, traumatic, vascular, or anoxic aetiology. The assessment will rely on neuropsychological instruments as well as on Magnetic Resonance Imaging (MRI) and Computed Tomography (CT) evidence. Exclusion criteria will be: presence of pacemaker or metallic cerebral implant, craniolacunia, ventricular peritoneal shunt, age <18 years, auditory injury as evaluated by anamnesis, history of neurological and psychiatric disease, previous stroke, use of alcohol or drugs, premorbid dementia. As recommended, a period of observation will be conducted for one week [46] twice a day by four independent examiners (physician, neuropsychologist, speech therapist and physiotherapist).

Before the enrolment, patients will undergo neurophysiological recordings, namely Brainstem Auditory Evoked Potentials (BAEP) to assess the integrity of the central auditory pathway. Patients will be divided according to the brain aetiology of their pathological condition. Caregivers will also be assessed to investigate the emotional and psychological burden and psychophysiological condition.

### Ethical considerations

Written consent will be obtained from all family caregivers in accordance with regulations.

The study protocol has been reviewed and approved by the Ethics Committee of ICS Maugeri Institute (prot. n. 345). The study will be conducted in accordance with the principles of the Declaration of Helsinki. Moreover, the protocol has been registered (Clinicaltrial.gov NCT05706831). Any changes to the protocol will be submitted to the ethics committee for approval.

Each patient entering the project will be assigned with a unique identifier and the code for linking private information to the identifier is securely stored at the Clinic. Identifiable information is restricted to authorised investigators from Maugeri Clinic. All outcome data are anonymised right after acquisition and then transferred to a password-protected folder on a server.

This protocol is a part of a more extended randomized cross-over intervention trial which includes also stroke patients stratified in patients with post-stroke aphasia and patients with post-stroke neglect. In this protocol paper, we focus only on patients with DOC.

### Sample size determination

The effect size to be expected is uncertain as no study so far has implemented a combined intervention of music and electrical stimulation in DOC patients. We assume it to be in the medium-to large range (i.e., Cohen’s d between 0.5 and 0.8). We aim to achieve 80% power (*f* = 0.30) in a repeated-measures analysis of variance (*a* = 0.05, power (1-b) = 0.80) with condition as between-subject factor (tES combined with music stimulation vs. sham stimulation, and noise vs. music stimulation and sham stimulation), and time (pre-intervention, post-intervention and post-placebo), as within-subject factor. Power calculations (using G* Power 3) indicate that a valid sample size of *n* = 10 per group will result in 80% statistical power if the effect is *d* = 0.68, which is in the medium-to-large range. This results in a total sample size of *n* = 30.

### Intervention

#### Brain stimulation

tES will be applied for 20 minutes every day, once a day, 5 times a week, for 2 weeks. During the first week, patients will be administered the real (*verum*) stimulation, while in the other week *sham* will be administered (the administration order will be counterbalanced across participants). Position of electrodes will be adjusted according to the clinical condition of the patient. In the *verum* condition, a direct current of 2 mA (anodal tDCS) will be delivered through a BrainStim battery driven electric stimulator in the target area of the brain, with the cathode on the contralateral arm. In the sham condition, the anode will deliver the direct current only for the first 20 sec. (i.e., 20 sec. rump-up).

#### Music stimulation

Music stimulation will consist of passive listening to 8 musical tracks. Four tracks will be chosen based on the musical preferences of each patient. Preferences will be derived from a musical anamnesis, namely a structured interview with the caregiver to know the patient’s lifestyle history and musical taste, conducted by the neuropsychologist. The outcome of this interview will help to tailor an individual treatment with familiar and self-relevant music, as previously done [47, 48]. The other four tracks are fixed and chosen by us based on previous literature: Adios Nonino by A. Piazzolla (used in several brain imaging studies); the Prelude in C major by J. S. Bach and the Prelude in C minor from the “Well-tempered Clavier”–I vol.; and a “atonal” version of the C major Prelude (also from the “Well-tempered Clavier”) where the original interval relationships between each note have been altered while maintaining the ascending/descending trend and temporal values of the notes. The two preludes and the atonal version have been used in previous neurophysiological and behavioural studies of auditory memory in healthy and elderly adults [49–52]. For optimising the stimulus variation, the eight tracks will be organized in playlists always alternating based on familiarity (the favourite song alternates a fixed one), on acoustic features (e.g., a track containing lyrics alternates an instrumental one) and emotional content (an arousing track alternates a relaxing one).

All patients will undergo standard physiotherapy rehabilitation treatment, according to the hospital guidelines, for one hour a day, 5 days a week. In addition, most of these patients are treated with specific drug therapy (such as, amantadine, citicoline, dopamine, etc.) in order to increase the level of consciousness. During data analysis these aspects will be considered and treated as necessary. Specifically, drug treatment and doses will be inserted as confounding factors and covariates in the ANOVAs.

### Experimental design and procedures

The experimental paradigm is one of the main strengths of the study, as a randomized cross-over design will be used in this trial, including a treatment condition with electric brain stimulation combined with music listening and a placebo condition with sham brain stimulation combined with noise listening. The randomization of the participants to be included in each group will be done using the software “Research Randomizer” (www.randomizer.org). The randomization procedure will be performed by an independent researcher unrelated to the study. The randomization file will be saved in a password-protected online database. This independent researcher will enrol participants and will assign the patients to intervention groups. Both patients and examiner will be fully blinded.

**Fig 1:**
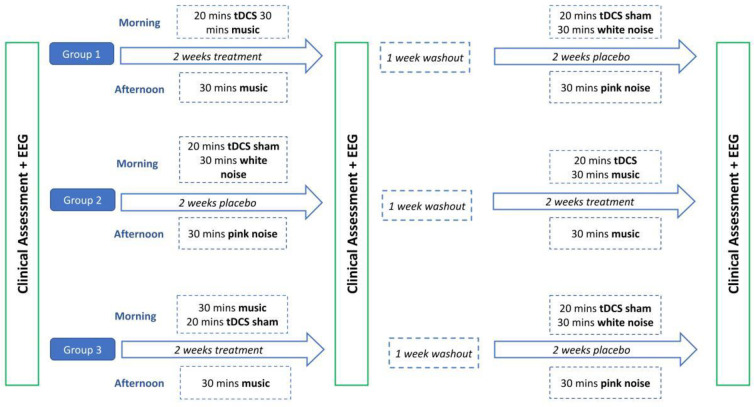
Study flow diagram displaying interventions and assessments.

Assessment times of outcome measures will be conducted at admission (T_0_), after the first weeks of post-intervention or post-placebo (T_1_), and after the second two weeks of post-intervention or post-placebo (T_2_) (Fig 1). Patients will be randomized into three groups depending on the order of the stimulation or placebo conditions:

1. Stimulation-Placebo: Patients receiving tES combined with music stimulation for 2 weeks, 1 week of wash out, then sham stimulation combined with noise (placebo) for other 2 weeks.
2. Placebo-stimulation (combined tES and music): Patients receiving sham stimulation and noise (placebo) for the first 2 weeks, then 1 week wash out, and finally tES and music stimulation for other 2 weeks.
3. Music-placebo: Patients receiving music stimulation and sham stimulation for 2 weeks, 1 week of wash out, then sham stimulation and noise (placebo) for another 2 weeks.

For the treatment condition, music stimulation, lasting overall around 30 minutes, will be provided by headphones for two weeks in a row every day (except weekends) twice a day: in the morning and in the afternoon. In the morning only, this music-listening treatment will be preceded by brain stimulation with tES applied in the hemisphere contralateral to the lesion (position of electrodes adjusted according to clinical condition of the patient). The placebo condition will have the same temporal course of the treatment condition, but, instead of music, patients will listen to white noise for 30 minutes in the morning, and pink noise for 30 minutes in the afternoon and only during the morning sessions, sham (current induction for 10 minutes ramp up and 10 minutes ramp down) in the hemisphere contralateral to the lesion will be delivered. Between the two conditions, one week of washout is planned.

Patients who will develop relevant clinical condition, such as respiratory problems, during the period of protocol, will be excluded from the study.

### Neurophysiological, neuropsychological and clinical primary outcomes

For obtaining the neurophysiological primary outcome measures, we will compute the power spectra of frequency bands from continuous EEG to assess the residual neuroplastic processes existing in DOC state, and we will then evaluate the putative modulation of these processes by the combined intervention of music and brain stimulation in a randomized cross-over design. EEG data will be acquired using a Galileo Mizar Plus medical device including 26 monopolar electrode channels, 16 bipolar electrode channels and 10 additional channels used for EEG recordings.

For the EEG measures, we will use the following paradigms:

1. Rest for 10 minutes with annotation on whether patients keep eyes open or closed for measuring the power spectra of frequency bands from the continuous EEG.
2. Free listening to the morning musical playlist as used in the music stimulation condition for measuring the power spectra of frequency bands and the evoked N100 responses from the continuous EEG.

For assessing the clinical conditions of DOC patients, the following scales will be used.

1. Glasgow Outcome Scale–Extended (GOS-E) [53]: A global scale for functional outcome that rates patient status in eight categories according to the disability.
2. Coma Recovery Scale—Revised (CRS-R) [54]: A tool which includes the current diagnostic criteria for coma, vegetative state, and the MCS, and allows the patient to be assigned to the most appropriate diagnostic category.
3. Disability Rating Scale (DRS) [55]: A scale that evaluates the functional changes in a rehabilitation setting. The DR Scale consists of 8 items divided into 4 categories: Arousal and awareness; cognitive ability to handle self-care functions; physical dependence upon others; psychosocial adaptability for work, housework, or school.
4. Rancho Levels of Cognitive Functioning (LCF) [56]: A scale that assesses cognitive functioning in post-coma patients, classifying them in one of eight level: No response; Generalized; Localized; Confused-agitated; Confused, inappropriate, non-agitated; Confused-appropriate; Automatic-appropriate; Purposeful-appropriate.

### Psychological and clinical secondary outcomes

We will also evaluate the impact of our intervention on caregiver’s burden and psychological distress by means of the following questionnaires for caregiver’s patients:

1. Beck Depression Inventory-II [57, 58]: A self-administered questionnaire to assess severity of depressive symptom, composed by 21 items to be evaluated on a 0–3 scale;
2. State-Trait anxiety inventory [59, 60]: A self -reported questionnaire to assess level of anxiety, both state and trait anxiety;
3. Psychophysiological Questionnaire/reduced form (CBA 2.0) [61]: A questionnaire composed by 30 items to assess the stress level and the frequency of subject’s psychophysiological reactions;
4. Prolonged grief disorder-12 [62, 63]: A questionnaire to evaluate the presence of separation distress and cognitive, behavioural or emotional symptoms at least for six months after the disease onset of a loved person;
5. Family strain questionnaire [64]: A questionnaire to assess perceived caregiving related problems;
6. World Health Organization–Quality of Life [65, 66]: A questionnaire evaluating QOL in four domains: physical, psychological, social and environment relations.

### Data analysis

The SPSS 23.0 statistical software will be used for the data analysis. All the statistical hypotheses will be tested by two-side test, with statistically significant test level set at 0.05 and the confidence interval estimation to 95%.

The aim of our study is to evaluate the effects of the treatments in the various patient groups on the primary and secondary outcomes. Descriptive analysis of the data will allow us to identify the characteristics of the groups. Comparison of demographic and clinical characteristics of the groups will be performed using one-way ANOVA for continuous variables and the Chi-square test for categorical variables. Considering the crossover and longitudinal aspects, primary analyses will focus on behavioural changes at group level, comparing the treatments. Moreover, at the individual level, we will analyse the differences between pre-treatments and post treatments data.

As for the primary outcomes, we will measure the effects of the intervention on the following measures (two neurophysiological and two clinical and behavioral): (i) power spectra of frequency band extracted from the EEG recordings; (ii) auditory evoked responses, extracted from the EEG recordings; (iii) behavioural responses of DOC patients, measured through CRS-R.

Baseline characteristics and carryover effect will be analysed between the three sequences using ANOVA and chi-square test. By including the order of intervention as a covariate, we aim to determine whether the sequence in which participants receive the interventions impacts the observed outcomes. To assess the presence and magnitude of the washout effect, we plan to incorporate appropriate statistical methods into our analysis, such as comparing outcomes during the washout period to those observed during active treatment phases. To this end, we will use repeated measures ANOVA considering time (before, during, and after the washout) as the independent variable, with behavioral measurements as the dependent variables.

For the secondary outcome we will measure the effects of the treatments on caregivers’ depression, anxiety, psychophysiological symptoms, grief, strain and quality of life in order to evaluate the effectiveness in reducing the caregiver’s burden and psychological distress. Repeated-measures analysis of variance, as well as post-hoc paired *t*-tests will be conducted to assess the effects of the treatments over time on N1, P1, and P2 amplitudes and latencies, as well as level of consciousness. All these primary outcome variables will be analysed separately, using parametric or non-parametric tests for continuous and categorical variables respectively.

For the behavioural outcome, we expect an improvement of level of consciousness, as measured by CRS-R.

For the caregiver’s burden, we expect a positive effect of this new treatment protocol on caregiver’s wellbeing, with a decrease of psychological distress.

For neurophysiological outcome measures, we expect to obtain in DOC patients an increased EEG power spectrum in slow frequency bands, following a previous study [67] showing a generalized slowing in the theta or delta range associated with a significantly diminished α power. Power spectrum analysis is one of the standard methods used for quantification of the EEG. The power spectrum, namely the power spectral density, reflects the ‘frequency content’ of the signal or the distribution of signal power over frequency [68].

For the computation of the auditory evoked responses, the average EEG will be measured time-locked to the sound onset events in the music stimuli, after rejecting any residual noise exceeding 100 μV. P1 (fronto-central positive potential with ~50 ms latency), N1 (fronto-central negative potential with ~100 ms latency), and P2 (fronto-central positive potential with ~200 ms latency) amplitudes and latencies will be measured at the Fz and Cz electrodes [69], and compared pre- and post- intervention.

To reach the goal of obtaining the above-described neurophysiological outcome measures, we will first pre-process the EEG data as follows. Data will be filtered to remove low frequencies not originating from the brain and to control for interference of the electric current, by means of a generic FIR filter, set up as a bandpass filter, with low cut-off at 0.5 Hz and high cut-off at 45 Hz (because of 50 Hz power line noise). Cerebral signals contaminated by artifacts will be identified and removed. Moreover, independent component analysis (ICA) will be performed. ICA algorithms will be used to detect and isolate artifacts, such as eye blinks, eye saccades, hearth, muscle, line noise, channel noise and others. In this way, the original signal will be decomposed into independent components: we will individuate and discard the components that picked up the artifacts activities and reconstruct a clear signal by using only the remaining components [70]. Further pre-processing steps will include epoch extraction, baseline removal, resampling, re-referencing to low temporal electrodes, and filtering.

To investigate whether slow neural oscillations are reduced and fast neural oscillations are increased post-treatment, the continuous EEG will first be divided into 2-seconds segments, transformed into the frequency domain with fast Fourier transform, inspected for myogenic artifact segments that will be rejected, and then averaged across the segments [71]. Any further exploratory analyses will be specified in the future publication of the findings of this study. Missing data will be treated according to Molenberghs et al. [72].

We will evaluate the music and electrical intervention effects on power spectra of frequency bands measuring EEG at rest and during music listening at T0 (pre-intervention), T1 (post-intervention) and T2 (post-placebo). To this end, we will compare the power spectra between rest and music conditions and across time. The ratio of fast (8–30 Hz, alpha and beta) to slow (2–8 Hz, delta and theta) oscillation amplitude at the midline electrodes (Fz, Cz, Pz, Oz) will be measured [70] and compared pre- and post- intervention using t-tests with cluster-based corrections of the multiple comparison problem. In the case of non-normal distributions, there will be applied mixed-effect ANOVAs or non-parametric equivalents to the parametric tests.

### Dissemination

Results of this research will be presented at national and international conferences on topic and published in a peer-reviewed journal.

### Data monitoring and management

A data monitoring committee has not been necessary because the study is considered to be of minimal risk. The paper and pencil tests and questionnaire will be stored in a locked cabinet and will be entered in an electronic file. The access to these data will be permitted to the research members only. Any adverse events will be reported by filling in the appropriate form. The data that support the findings of this study will be openly available on Zenodo.

## Discussion

With this trial we present a novel clinical intervention combining music exposure with NIBS in DOC patients, also assessing the impact of this rehabilitation protocol on caregiver’s burden and psychological distress. Both music and NIBS have been largely proven effective in the realm of brain lesions. Nonetheless, this is the first study to propose a clinical protocol including a combined effect of these two intervention strategies among post-coma patients. Further strengths of the current protocol are the sample size and the use of a variety of objective measures, including neurophysiological ones such as EEG, to show the cognitive and functional improvement of DOC patients.

In relation to EEG recorded during music stimulation, our intervention is expected to produce two outcomes. The first consists of increased evoked responses amplitudes to sounds reflecting a residual plasticity of the auditory cortex in DOC patients after the music-based intervention combined with electric stimulation. Second, the continuous EEG is expected to display a decrease of power spectra of slow frequency bands and an increase in power for faster frequency bands. Overall, our ambitious aim of inducing and evaluating beneficial effects of music and brain stimulation on psychological and neurophysiological measures would represent a novel rehabilitation approach, not yet explored in DOC patients.

The experimental paradigm we designed is one of the major strengths of the study. Indeed, as RCTs provide the most reliable evidence on the effectiveness of interventions, a pivotal condition especially in clinical research, this protocol significantly contributes to shed light on the most appropriate characteristics for DOC-targeted interventions. As such, this study may help pave the way towards a more generalised and replicable protocol on DOC rehabilitation, as well as highlight the potential role of music on brain injury rehabilitation. In this sense, as our experimental design includes both preferred and non-preferred musical stimuli, this study will allow us to entangle the beneficial effects of music ascribable to its acoustic characteristics from those arising from its autobiographical content, yet unexplored in prior studies on DOC [27, 46]. A deeper understanding of musically-induced positive emotions in DOC patients might be central in sharpening cognitive stimulation processes and personal memory activation. This is particularly relevant for the clinical population we are considering as DOC can be related to damage of the dopaminergic system [26]. In other words, as rewarding music can activate the dopaminergic system via changes in the limbic system (i.e., [73]), the association between music stimulation and cognitive improvements in DOC might be mediated by musically-related positive emotions.

Another strength of this trial refers to its implications on the caregiver. Taking care of patients with DOC has a high impact on a caregiver’s social, economic and emotional condition. High levels of depression, anxiety and prolonged grief were reported both at admission both at follow-up in caregivers of DOC patients [74]. However, most music interventions studies have focused on caregivers of patients affected by dementia (for a review see [75]). As far as we know, there are no studies assessing the effects of music stimulation on DOC patients’ caregivers’ burden. We aim to fill this gap considering that music may be an efficient additional caring tool for caregivers for several reasons. First, in our study caregivers actively contribute to the intervention by suggesting patients’ musical preferences to the neuropsychologist and the experimental team. This direct involvement is likely to promote caregivers’ empowerment and increased control, as seen in previous studies (i.e., [76, 77]). Second, music-based interventions are generally associated with fostered feelings of inner strength, personal growth, increased resilience, social confidence and self-esteem amongst caregivers [76]. Therefore, our rehabilitation protocol could be valid and effective in relieving caregiver’s emotional symptoms.

We also acknowledge some limitations that should be considered for the interpretation of the results. First, since our experimental paradigm provides for tailored salient emotional stimuli (namely preferred music), individual differences on music reward sensitivity might influence the elicited responses. Indeed, prior evidence suggested that individual music reward sensitivity affects both physiological (skin conductance) and neural (mainly dopaminergic mesolimbic reward system) responses (see [78, 79]). A second potential limitation is related to the possible overestimation of the treatment effect size, due to the lack of studies performing this multimodal intervention on DOC patients. Accordingly, in the event of preliminary analyses revealing an effect size significantly lower than predicted, we will consider an extension of the population size. Lastly, given the nature of our target population and intervention, which involves specialised healthcare professionals and specifical equipment, we are unable to blind participants and assessors. All in all, this protocol has a high clinical applicability, it is easy to offer, and it is very motivating for the patients and the caregivers. We, therefore, believe that our findings may help to open new paths in the development of new specific DOC rehabilitation approaches.

## Supporting information

S1 Checklist SPIRIT 2013 checklist: Recommended items to address in a clinical trial protocol and related documents*. (DOC)

S1 File (DOCX)

S2 File (DOCX)
