# Supplementary material for: Musical and electrical stimulation as intervention in disorder of consciousness (DOC) patients: A randomised cross-over trial
Source: PLoS One. 2024 May 31;19(5):e0304642. doi: 10.1371/journal.pone.0304642 (PMC11142721; doi:10.1371/journal.pone.0304642)
Supplement: S1 Checklist — (DOC) [file pone.0304642.s001.doc]

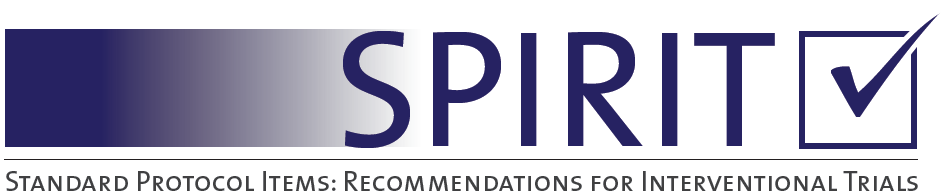


SPIRIT 2013 Checklist: Recommended items to address in a clinical trial protocol and related documents*

| Section/item | ItemNo | Description |
| --- | --- | --- |
| **Administrative information** | | |
| Title | 1 | MUSIC THERAPY AND TRANSCRANIAL ELECTRICAL STIMULATION  FOR TREATING NEUROLOGICAL DISEASES |
| Trial registration | 2a | ClinicalTrials.gov identifier: NCT05706831, registered on January 30, 2023. |
|  | 2b | --- |
| Protocol version | 3 | Vers n.4 19/05/2022 |
| Funding | 4 | NA |
| Roles and responsibilities | 5a | Simona Spaccavento^1^, Elvira Brattico^2^, Davide Rivolta^2^ & Ernesto Losavio^1^  ^1^ ^1^Istituti Clinici Scientifici Maugeri IRCCS, Institute of Bari, Via Generale Bellomo, 73/75 - 70124 Bari, Italy.  ^2^ Department of Education, Psychology, Communication, University of Bari Aldo Moro, Italy |
|  | 5b | NA |
|  | 5c | NA |
|  | 5d | NA |
| Introduction |  |  |
| Background and rationale | 6a | Disorders of consciousness (DOC), i.e., unresponsive wakefulness syndrome (UWS) or vegetative state (VS) and minimally conscious state (MCS), are conditions that can arise from severe brain injury, inducing widespread functional changes. Given the damaging implications resulting from these conditions, there is an increasing need for rehabilitation treatments aimed at enhancing the level of consciousness, the quality of life, and creating new recovery perspectives for the patients. Music may represent an additional rehabilitative tool in contexts where cognition and language are severely compromised, such as among DOC patients. A further type of rehabilitation strategies for DOC patients consists of non-invasive brain stimulation techniques (NIBS), including transcranial electrical stimulation (tEs), affecting neural excitability and promoting brain plasticity. |
|  | 6b | NA |
| Objectives | 7 | With this study protocol, we propose a novel rehabilitation approach for DOC patients combining music-based intervention and non-invasive brain stimulation in neurological patients. The main objectives are (1) to assess the residual neuroplastic processes in DOC patients exposed to music, (2) to determine the putative neural modulation and the clinical outcome in DOC patients of non-pharmacological strategies, i.e., electric (tES) and music stimulation, and (3) to evaluate the putative positive impact of this intervention on caregiver’s burden and psychological distress. |
| Trial design | 8 | A randomized cross-over design will be used in this trial, including a treatment condition with electric brain stimulation combined with music listening and a placebo condition with sham brain stimulation combined with noise listening. The randomization of the participants to be included in each group will be done using the software “Research Randomizer” ([www.randomizer.org](http://www.randomizer.org/)). |
| Methods: Participants, interventions, and outcomes | | |
| Study setting | 9 | All patients consecutively admitted to the Neurorehabilitation Unit at the ICS Maugeri (Bari, Italy) with Vegetative State (VS) or minimally conscious state (MCS) will be assessed |
| Eligibility criteria | 10 | Inclusion criteria will be the following: clinical diagnosis of VS or MCS according to standardised clinical diagnostic criteria, traumatic, vascular or anoxic aetiology. Exclusion criteria will be: presence of pacemaker or metallic cerebral implant, craniolacunia, ventricular peritoneal shunt, age <18 years, auditory injury as evaluated by anamnesis, history of neurological and psychiatric disease, previous stroke, use of alcohol or drugs, premorbid dementia. |
| Interventions | 11a | *Brain stimulation*. The tES will be applied for 20 minutes every day, once a day, 5 times a week, for 2 weeks. During one week patients will be administered the real (*verum*) stimulation, while in the other week the sham will be administered (the administration order will be counterbalanced across participants). Position of electrodes will be adjusted according to the clinical condition of the patient. In the *verum* condition, a direct current of 2 mA (anodal tDCS) will be delivered through a BrainStim battery driven electric stimulator in the target area of the brain, with the cathode on the contralateral arm. In the sham condition, the anode will deliver the direct current only for the first 20 sec. (i.e., 20 sec. rump-up).  *Music stimulation.* The music stimulation will consist of passive listening to 8 musical tracks. Four tracks will be chosen based on the musical preferences of each patient. Preferences will be derived from a musical anamnesis, namely a structured interview with the caregiver to know the patient’s lifestyle history and musical taste, conducted by the neuropsychologist. The outcome of this interview will help to tailor an individual treatment with familiar and self-relevant music, as previously done. The other four tracks are fixed and chosen by us based on previous literature: Adios Nonino by A. Piazzolla (used in several brain imaging studies); the Prelude in C major by J. S. Bach and the Prelude in C minor from the “Clavicembalo Ben temperato” – I vol.; and a “de-tonalized” version of the C major prelude where the original interval relationships between each note have been altered while maintaining the ascending/descending trend and temporal values of the notes. The two preludes and the de-tonalised version have been used in previous brain imaging studies of memory. For optimising the stimulus variation, the eight tracks will be organized in playlists always alternating based on familiarity (the favourite song alternates a fixed one), on acoustic features (e.g., a track containing lyrics alternates an instrumental one) and emotional content (an arousing track alternates a relaxing one). |
|  | 11b | Patients who will develop relevant clinical condition, such as respiratory problems, during the period of protocol, will be excluded from the study. |
|  | 11c | --- |
|  | 11d | --- |
| Outcomes | 12 | We will collect power spectra of frequency bands from continuous EEG to assess the residual neuroplastic processes existing in DOC state. In addition, we will evaluate the putative modulation of these processes by a combined intervention of music and brain stimulation. EEG data will be acquired using a Galileo Mizar Plus medical device including 26 monopolar electrode channels, 16 bipolar electrode channels and 10 additional channels used for EEG recordings.  For obtaining neurophysiological primary outcome measures, we will use the following paradigms:   1. Rest for 10 minutes with annotation on whether patients keep eyes open or closed for measuring the power spectra of frequency bands from the continuous EEG. 2. Free listening to the morning musical playlist as used in the music stimulation condition for measuring the power spectra of frequency bands and the evoked N100 responses from the continuous EEG.   For assessing the clinical conditions of DOC patients, the following scales will be used.   - - - 1. Glasgow Outcome Scale – Extended (GOS-E) [53]: A global scale for functional outcome that rates patient status in eight categories according to the disability.       2. Coma Recovery Scale - Revised (CRS-R) [54]: A tool which includes the current diagnostic criteria for coma, vegetative state, and the MCS, and allows the patient to be assigned to the most appropriate diagnostic category.       3. Disability Rating Scale (DRS) [55]: A scale that evaluates the functional changes in a rehabilitation setting. The DR Scale consists of 8 items divided into 4 categories: Arousal and awareness; cognitive ability to handle self-care functions; physical dependence upon others; psychosocial adaptability for work, housework, or school.       4. Rancho Levels of Cognitive Functioning (LCF) [56]: A scale that assesses cognitive functioning in post-coma patients, classifying them in one of eight level: No response; Generalized; Localized; Confused-agitated; Confused, inappropriate, non-agitated; Confused-appropriate; Automatic-appropriate; Purposeful-appropriate.   *Psychological and clinical secondary outcomes*  We will also evaluate the impact of our intervention on caregiver’s burden and psychological distress by means of the following questionnaires for caregiver’s patients:  Beck Depression Inventory-II [57], [58]: A self-administered questionnaire to assess severity of depressive symptom, composed by 21 items to be evaluated on a 0-3 scale;  State-Trait anxiety inventory [59], [60]: A self -reported questionnaire to assess level of anxiety, both state and trait anxiety;  Psychophysiological Questionnaire /reduced form (CBA 2.0) [61]: A questionnaire composed by 30 items to assess the stress level and the frequency of subject’s psychophysiological reactions;  Prolonged grief disorder-12 [62], [63]: A questionnaire to evaluate the presence of separation distress and cognitive, behavioural or emotional symptoms at least for six months after the disease onset of a loved person;  Family strain questionnaire [64]: A questionnaire to assess perceived caregiving related problems;  World Health Organization – Quality of Life [65], [66]: A questionnaire evaluating QOL in four domains: physical, psychological, social and environment relations.  Assessment times of outcome measures will be conducted at admission (T_0_), after the first weeks of post-intervention or post-placebo (T_1_), and after the second two weeks of post-intervention or post-placebo (T_2_). |
| Participant timeline | 13 | A randomized cross-over design will be used in this trial, including a treatment condition with electric brain stimulation combined with music listening and a placebo condition with sham brain stimulation combined with noise listening. The randomization of the participants to be included in each group will be done using the software “Research Randomizer” ([www.randomizer.org](http://www.randomizer.org/)).  Assessment times of outcome measures will be conducted at admission (T_0_), after the first weeks of post-intervention or post-placebo (T_1_), and after the second two weeks of post-intervention or post-placebo (T_2_). Patients will be randomized into three groups depending on the order of the stimulation or placebo conditions:   1. Stimulation-Placebo: Patients receiving tES combined with music stimulation for 2 weeks, 1 week of wash out, then sham stimulation combined with noise (placebo) for other 2 weeks; 2. Placebo-stimulation: Patients receiving sham stimulation and noise (placebo) for the first 2 weeks, then 1 week wash out, and finally tES and music stimulation for other 2 weeks; 3. Music-placebo: Patients receiving music stimulation and sham stimulation for 2 weeks, 1 week of wash out, then sham stimulation and noise (placebo) for another 2 weeks.   For the treatment condition, music stimulation, lasting overall around 30 minutes, will be provided by headphones for two weeks in a row every day (except weekends) twice a day: in the morning and in the afternoon. In the morning only, this music-listening treatment will be preceded by brain stimulation with tES applied in the hemisphere contralateral to the lesion (position of electrodes adjusted according to clinical condition of the patient). The placebo condition will have the same temporal course of the treatment condition, but, instead of music, patients will listen to white noise for 30 minutes in the morning, and pink noise for 30 minutes in the afternoon and only during the morning sessions, sham (current induction for 10'' ramp up and 10'' ramp down) for 20 minutes in the hemisphere contralateral to the lesion will be delivered. Between the two conditions, one week of washout is planned. (see Figure) |
| Sample size | 14 | The effect size to be expected is uncertain as no study so far has implemented a combined intervention of music and electrical stimulation in DOC patients. We assume it to be in the medium-to large range (i.e., Cohen’s d between 0.5 and 0.8). We aim to achieve 80% power (*f* = 0.30) in a repeated-measures analysis of variance (*a* = 0.05, power (1-b*)* = 0.80) with condition as between-subject factor (tES combined with music stimulation vs. sham stimulation, and noise vs. music stimulation and sham stimulation), and time (pre-intervention, post-intervention and post-placebo), as within-subject factor. Power calculations (using G* Power 3) indicate that a valid sample size of *n* = 10 per group will result in 80% statistical power if the effect is *d* = 0.68, which is in the medium-to-large range. This results in a total sample size of *n* = 30. |
| Recruitment | 15 | Our hospital is a reference centre for patients with severe acquired brain injury; an information will be sent to the acute hospitals from which patients come with the aim of informing them of the research project. |
| **Methods: Assignment of interventions (for controlled trials)** | | |
| Allocation: |  |  |
| Sequence generation | 16a-b-c | A randomized cross-over design will be used in this trial, including a treatment condition with electric brain stimulation combined with music listening and a placebo condition with sham brain stimulation combined with noise listening. The randomization of the participants to be included in each group will be done using the software “Research Randomizer” ([www.randomizer.org](http://www.randomizer.org/)). The randomization procedure will be performed by an independent research unrelated to the study. The randomization file will be saved in a password-protected online database. This independent research will enrol partecipants and will assign the patients to intervention groups. |
| Allocation concealment mechanism | 16b | --- |
| Implementation | 16c | --- |
| Blinding (masking) | 17a | Patients and examiner will be fully blinded. |
|  | 17b | --- |
| **Methods: Data collection, management, and analysis** | | |
| Data collection methods | 18a | We will collect power spectra of frequency bands from continuous EEG to assess the residual neuroplastic processes existing in DOC state. In addition, we will evaluate the putative modulation of these processes by a combined intervention of music and brain stimulation. EEG data will be acquired using a Galileo Mizar Plus medical device including 26 monopolar electrode channels, 16 bipolar electrode channels and 10 additional channels used for EEG recordings.  For obtaining neurophysiological primary outcome measures, we will use the following paradigms:   1. Rest for 10 minutes with annotation on whether patients keep eyes open or closed for measuring the power spectra of frequency bands from the continuous EEG. 2. Free listening to the morning musical playlist as used in the music stimulation condition for measuring the power spectra of frequency bands and the evoked N100 responses from the continuous EEG.   For assessing the clinical conditions of DOC patients, the following scales will be used.   - - - 1. Glasgow Outcome Scale – Extended (GOS-E) [53]: A global scale for functional outcome that rates patient status in eight categories according to the disability.       2. Coma Recovery Scale - Revised (CRS-R) [54]: A tool which includes the current diagnostic criteria for coma, vegetative state, and the MCS, and allows the patient to be assigned to the most appropriate diagnostic category.       3. Disability Rating Scale (DRS) [55]: A scale that evaluates the functional changes in a rehabilitation setting. The DR Scale consists of 8 items divided into 4 categories: Arousal and awareness; cognitive ability to handle self-care functions; physical dependence upon others; psychosocial adaptability for work, housework, or school.       4. Rancho Levels of Cognitive Functioning (LCF) [56]: A scale that assesses cognitive functioning in post-coma patients, classifying them in one of eight level: No response; Generalized; Localized; Confused-agitated; Confused, inappropriate, non-agitated; Confused-appropriate; Automatic-appropriate; Purposeful-appropriate.   *Psychological and clinical secondary outcomes*  We will also evaluate the impact of our intervention on caregiver’s burden and psychological distress by means of the following questionnaires for caregiver’s patients:  Beck Depression Inventory-II [57], [58]: A self-administered questionnaire to assess severity of depressive symptom, composed by 21 items to be evaluated on a 0-3 scale;  State-Trait anxiety inventory [59], [60]: A self -reported questionnaire to assess level of anxiety, both state and trait anxiety;  Psychophysiological Questionnaire /reduced form (CBA 2.0) [61]: A questionnaire composed by 30 items to assess the stress level and the frequency of subject’s psychophysiological reactions;  Prolonged grief disorder-12 [62], [63]: A questionnaire to evaluate the presence of separation distress and cognitive, behavioural or emotional symptoms at least for six months after the disease onset of a loved person;  Family strain questionnaire [64]: A questionnaire to assess perceived caregiving related problems;  World Health Organization – Quality of Life [65], [66]: A questionnaire evaluating QOL in four domains: physical, psychological, social and environment relations.  Assessment times of outcome measures will be conducted at admission (T_0_), after the first weeks of post-intervention or post-placebo (T_1_), and after the second two weeks of post-intervention or post-placebo (T_2_). |
|  | 18b | --- |
| Data management | 19 | A data monitoring committee has not been necessary because the study is considered to be of minimal risk. The paper and pencil tests and questionnaire will be stored in a locked cabinet and will be entered in an electronic file. The access to these data will be permitted to the research members only. |
| Statistical methods | 20a | The SPSS 23.0 statistical software will be used for the data analysis. All the statistical hypotheses will be tested by two-side test, with statistically significant test level set at 0.05 and the confidence interval estimation to 95%.  The aim of our study is to evaluate the effects of the treatments in the various patient groups on the primary and secondary outcomes. Descriptive analysis of the data will allow us to identify the characteristics of the groups. Comparison of demographic and clinical characteristics of the groups will be performed using one-way ANOVA for continuous variables and the Chi-square test for categorical variables. Considering the crossover and longitudinal aspects, primary analyses will focus on behavioural changes at group level, comparing the treatments.¸Moreover, at the individual level, we will analyse the differences between pre-treatments and post treatments data.  As for the primary outcomes, we will measure the effects of the intervention on the following measures (two neurophysiological and two clinical and behavioral): (i) power spectra of frequency band extracted from the EEG recordings; (ii) auditory evoked responses, extracted from the EEG recordings; (iii) behavioural responses of DOC patients, measured through CRS-R.  Baseline characteristics and carryover effect will be analysed between the three sequences using ANOVA and chi-square test. By including the order of intervention as a covariate, we aim to determine whether the sequence in which participants receive the interventions impacts the observed outcomes. To assess the presence and magnitude of the washout effect, we plan to incorporate appropriate statistical methods into our analysis, such as comparing outcomes during the washout period to those observed during active treatment phases. To this end, we will use repeated measures ANOVA considering time (before, during, and after the washout) as the independent variable, with behavioral measurements as the dependent variables.  For the secondary outcome we will measure the effects of the treatments on caregivers’ depression, anxiety, psychophysiological symptoms, grief, strain and quality of life in order to evaluate the effectiveness in reducing the caregiver’s burden and psychological distress. Repeated-measures analysis of variance, as well as post-hoc paired t-tests will be conducted to assess the effects of the treatments over time on N1, P1, and P2 amplitudes and latencies, as well as level of consciousness. All these primary outcome variables will be analysed separately, using parametric or non-parametric tests for continuous and categorical variables respectively.  For the behavioural outcome, we expect an improvement of level of consciousness, as measured by CRS-R.  For the caregiver’s burden, we expect a positive effect of this new treatment protocol on caregiver’s wellbeing, with a decrease of psychological distress.  For neurophysiological outcome measures, we expect to obtain in DOC patients an increased EEG power spectrum in slow frequency bands, following a previous study [67] showing a generalized slowing in the theta or delta range associated with a significantly diminished α power. Power spectrum analysis is one of the standard methods used for quantification of the EEG. The power spectrum, namely the power spectral density, reflects the ‘frequency content’ of the signal or the distribution of signal power over frequency [68].  For the computation of the auditory evoked responses, the average EEG will be measured time-locked to the sound onset events in the music stimuli, after rejecting any residual noise exceeding 100 μV. P1 (fronto-central positive potential with ~50 ms latency), N1 (fronto-central negative potential with ~100 ms latency), and P2 (fronto-central positive potential with ~200 ms latency) amplitudes and latencies will be measured at the Fz and Cz electrodes [69], and compared pre- and post- intervention.  To reach the goal of obtaining the above-described neurophysiological outcome measures, we will first pre-process the EEG data as follows. Data will be filtered to remove low frequencies not originating from the brain and to control for interference of the electric current, by means of a generic FIR filter, set up as a bandpass filter, with low cut-off at 0.5 Hz and high cut-off at 45 Hz (because of 50 Hz power line noise). Cerebral signals contaminated by artifacts will be identified and removed. Moreover, independent component analysis (ICA) will be performed. ICA algorithms will be used to detect and isolate artifacts, such as eye blinks, eye saccades, hearth, muscle, line noise, channel noise and others. In this way, the original signal will be decomposed into independent components: we will individuate and discard the components that picked up the artifacts activities and reconstruct a clear signal by using only the remaining components [70]. Further pre-processing steps will include epoch extraction, baseline removal, resampling, re-referencing to low temporal electrodes, and filtering. |
|  | 20b | To investigate whether slow neural oscillations are reduced and fast neural oscillations are increased post-treatment, the continuous EEG will first be divided into 2-seconds segments, transformed into the frequency domain with fast Fourier transform, inspected for myogenic artifact segments that will be rejected, and then averaged across the segments [71]. Any further exploratory analyses will be specified in the future publication of the findings of this study. Missing data will be treated according to Molenberghs et al. [72].  We will evaluate the music and electrical intervention effects on power spectra of frequency bands measuring EEG at rest and during music listening at T0 (pre-intervention), T1 (post-intervention) and T2 (post-placebo). To this end, we will compare the power spectra between rest and music conditions and across time. The ratio of fast (8-30 Hz, alpha and beta) to slow (2-8 Hz, delta and theta) oscillation amplitude at the midline electrodes (Fz, Cz, Pz, Oz) will be measured 70 and compared pre- and post- intervention using t-tests with cluster-based corrections of the multiple comparison problem. In the case of non-normal distributions, there will be applied mixed-effect ANOVAs or non-parametric equivalents to the parametric tests |
|  | 20 c | Missing data will be treated according to Molenberghs et al. |
| **Methods: Monitoring** | | |
| Data monitoring | 21a | A data monitoring committee has not been necessary because the study is considered to be of minimal risk. The paper and pencil tests and questionnaire will be stored in a locked cabinet and will be entered in an electronic file. The access to these data will be permitted to the research members only. |
|  | 21b | --- |
| Harms | 22 | Any adverse events will be reported by filling in the appropriate form |
| Auditing | 23 | --- |
| Ethics and dissemination | | |
| Research ethics approval | 24 | The study protocol has been reviewed and approved by the Ethics Committee of ICS Maugeri Institute (prot. n. 345). The study will be conducted in accordance with the principles of the Declaration of Helsinki. Moreover, the protocol has been registered (Clinicaltrial.gov NCT05706831) |
| Protocol amendments | 25 | Any changes to the protocol will be submitted to the ethics committee for approval. |
| Consent or assent | 26a | Written consent will be obtained from all family caregivers in accordance with regulations. The study protocol has been reviewed and approved by the Ethics Committee of ICS Maugeri Institute (prot. n. 345). The study will be conducted in accordance with the principles of the Declaration of Helsinki. Moreover, the protocol has been registered (Clinicaltrial.gov NCT05706831). Each patient entering the project will be assigned with a unique identifier and the code for linking private information to the identifier is securely stored at the Clinic. Identifiable information is restricted to authorised investigators from Maugeri Clinic. All outcome data are anonymised right after acquisition and then transferred to a password-protected folder on a server. |
|  | 26b | NA |
| Confidentiality | 27 | Each patient entering the project will be assigned with a unique identifier and the code for linking private information to the identifier is securely stored at the Clinic. Identifiable information is restricted to authorised investigators from Maugeri Clinic. All outcome data are anonymised right after acquisition and then transferred to a password-protected folder on a server. |
| Declaration of interests | 28 | The authors have no financial or personal relationships with other people and organizations that could inappropriately influence their work. |
| Access to data | 29 | Access to the final trial dataset will be limited to the protocol authors |
| Ancillary and post-trial care | 30 | ---- |
| Dissemination policy | 31a | Results of this research will be presented at national and international conferences on topic and published in a peer-reviewed journal. |
|  | 31b | ---- |
|  | 31c | The protocol has been registered on clinicaltrials.gov |
| Appendices |  |  |
| Informed consent materials | 32 | --- |
| Biological specimens | 33 | NS |
