# Supplementary material for: Musical and electrical stimulation as intervention in disorder of consciousness (DOC) patients: A randomised cross-over trial
Source: PLoS One. 2024 May 31;19(5):e0304642. doi: 10.1371/journal.pone.0304642 (PMC11142721; doi:10.1371/journal.pone.0304642)
Supplement: S1 File — (DOCX) [file pone.0304642.s002.docx]

Prot. N. 345 C.E.

The Ethics Committee of the "John Paul II" Cancer Institute of Bari met in the Institute's Conference Room, in compliance with the provisions of the DPCM in force for the containment of the COVID-19 infection, on 27 June 2022 at 3.00 pm for the evaluation of the following protocol:

SUBJECT: Transmission of opinion Substantial amendment to the MUSIC THERAPY protocol: "Music therapy and transcranial electrical stimulation for treating neurological diseases" (Prot. 62/CE Maugeri) Responsible: Dr. Simona SPACCAVENTO

Documents presented and examined by the Ethics Committee:

o Communication of the amendment of 05.19.2022;

o Amended protocol.

After evaluating the documents indicated above, the Ethics Committee unanimously expresses a FAVORABLE opinion on the amendment in question. The opinion expressed above is limited exclusively to the versions cited and to the documentation presented. Any variation to the same must be compulsorily submitted to the opinion of this EC. All reports relating to adverse events, serious or unexpected, arising during the study that could affect the safety of the subjects, as well as any communication regarding the conclusion of the study or its possible interruption and any integration thereof must be communicated to the undersigned Ethics Committee.

We declare that the Components involved in any capacity in the trial in question and who therefore could have direct or indirect conflicts of interest have abstained from making a statement.
