# Supplementary material for: Musical and electrical stimulation as intervention in disorder of consciousness (DOC) patients: A randomised cross-over trial
Source: PLoS One. 2024 May 31;19(5):e0304642. doi: 10.1371/journal.pone.0304642 (PMC11142721; doi:10.1371/journal.pone.0304642)
Supplement: S2 File — (DOCX) [file pone.0304642.s003.docx]

**MUSIC THERAPY AND TRANSCRANIAL ELECTRICAL STIMULATION**

**FOR TREATING NEUROLOGICAL DISEASES**

**Introduction**

Acquired brain injury (ABI) includes a wide range of conditions caused by traumatic event, such as traumatic brain injury, by vascular event, such as ischemic or haemorrhagic stroke, by cerebral anoxia, toxic or metabolic insult.

All these neurological conditions can result in impairments in motor and cognitive functions as well as in emotional and neuropsychiatric symptoms that causing a significant level of disabilities and poor functional outcome.

A number of different rehabilitation techniques and treatments have been developed for the each of neuropsychological deficits (aphasia, neglect, attention and memory deficits, etc.)

Over the last years, music therapy has been used as a therapeutic tool in rehabilitation settings, including brain lesioned patients, with the aim to stimulate brain functions involved in movement, cognitive functions, such as language, memory, attention, etc., and emotions. Various studies (e.g., Sarkamo et al., 2008; Sarkamo et al., 2014; Chen et al., 2013; Leonardi et al., 2018) have evaluated the role of music interventions in brain-lesioned patients with specific cognitive deficits, such as aphasia, attention deficits and neglect. These authors found a significant effect of music therapy in improving cognitive deficits, considering music interventions effective in rehabilitative settings. Auditory stimuli can activate the right hemisphere in the brain which is dominant for sustained attention and those stimuli can influence spatial attention including unilateral visual neglect. Kang and Thaut (2019) had developed the Musical Neglect Training (MNT), a training for patients with unilateral visual neglect that uses active musical exercises in order to improve left visual field processing. Results demonstrated a positive effect of MNT in patients with chronic neglect.

The rehabilitation with the use of music can be efficacy especially in severe brain injury disease such as disorders of consciousness (DOC) for the treatment and rehabilitation of which very few options exist.

The idea is that music is a nonverbal auditory stimulus with emotional salience that could function as an alternative communication channel above all with patients with disorders of consciousness (DOC). Some scholars also view music listening as another kind of enriched sensory stimulation, making then music therapy a subtype of the cognitive rehabilitation strategies. Neuropsychological studies have shown that music increases arousal and attentions in these patients with significant cortical and autonomic nervous system activity (O’Kelly et al., 2013). Moreover, several studies reported physiological responses such as respiration and heart rate (Aldridge et al., 1990), and behavioural changes such as body movements, eye opening, tracking and orienting towards the therapist and source of music (Gustorff, 2002; Tamplin, 2000).

In DOC patients, one rehabilitation strategy (‘cognitive’) relies on sensorimotor regulation programs promoting recovery by the exposure to afferent sensory input. Sensory stimulation programs have been the most frequently applied treatment during patient’s neurorehabilitation (Tolle and Reimer, 2003). These programs are based on the idea that an enriched environment benefits brain plasticity and improves recovery of injured brains. Multisensory stimulation is also the basis of the so-called “basal stimulation”, which comprises somatosensory, vestibular, vibratory, auditory, tactile and visual stimuli during the nursing process (Kater, 1989).

Another group of rehabilitation strategies for brain-lesioned patients, including DOC patients, consists of *neuromodulation* aiming to alter the physiology of the neural circuits responsible for the behaviour under investigation. Among neuromodulation approaches there are pharmacological interventions (e.g., amantadine hydrochloride) (Giacino et al., 2012), deep brain stimulation and non-invasive brain stimulation techniques (NIB). NIB exists in various forms, albeit the most adopted in clinical settings is transcranial electrical stimulation (tEs). This technique consists in delivering a small (1-2 mA) current via two electrodes placed on the scalp. According to the stimulation parameters, tEs affects neural excitability and has plastic effects (mediated by NMDA-receptors) (Nitsche et al., 2003). Various tEs studies show improvements in clinical (e.g., GCS, CRS-R, cognitive, motor) and surrogate (i.e., EEG, TMS, fMRI) outcomes of people with DOC (albeit with more effects in MCSs than UWSs), thus suggesting that NIB can be a useful tool in the rehabilitation of clinical populations such as DOCs (Dimitri et al., 2017).

In the last years, tEs are used as a potential adjuvant treatment to improve symptoms in patients with hemispatial neglect after stroke (Salazar et al., 2018). The non-invasive brain stimulation is combined with other therapies such as prism adaptation, optokinetic training or cognitive therapy.

Many studies are evaluated the efficacy of TDCS in the rehabilitation of language disease in patients with aphasia, always together with language therapy (Marangolo, 2017).

The outcome of these two rehabilitation strategies in neurological patients are influenced above all by the sample size, the stage of disease (acute or chronic) and the corresponding setting (emergency room or long-term hospitalisation). Moreover, the methodology adopted in different tEs studies is characterized by a large variability, as concerns stimulation parameters, participant characteristics as well as the characteristics of associated rehabilitation treatment.

The aim of this study is to evaluate the efficacy of a novel rehabilitation protocol that would combine music therapy with non-invasive brain stimulation in neurological patients, assessing also the influence of this rehabilitation protocol on caregiver’s burden and psychological distress. The outcome measures will be clinical (neurologist’s observations) and neurophysiological (power spectra of slow frequency bands of brain oscillation and mismatch negativity responses to auditory stimulation paradigms) for DOC patients; neuropsychological and functional measures will be considered as outcome for patients with unilateral lesions. Subjective self-reports with standard psychological questionnaires for caregivers will be also considered.

**Materials and Methods**

Three groups of patients will be considered. The study will last 2 years.

1. **DOC Patients**

All VS or MCS patients consecutively admitted to our Neurorehabilitation Unit will be enrolled in this study. Inclusion criteria are age >18 years, no auditory injury as evaluated by anamnesis, no history of neurological and psychiatric disease. A period of observation is conducted for 1 week, twice a day by four independent examiners (physician, neuropsychologist, speech therapist and physiotherapist).

**Sample size determination**

We decided to test 30 DOC patients (*n* = 10 per experimental paradigm) as a power analysis using G∗Power 3 suggested that this sample size will be sufficient to achieve a medium effect (f = 0.25) in a repeated-measures analysis of variance (*a* = 0.05, power (1-*b*) = 0.80) with condition as the between-subject factor (tEs combined with music stimulation vs. sham stimulation and noise vs. music stimulation and sham stimulation) and time (pre-intervention, post-intervention and post-placebo) as the within-subject factor. In the case of dropouts, we will continue recruitment and repeat the randomization procedure until at least a number of 30 participants is collected.

**Outcome measures**

The following scales will be used:

- Electroencephalography (EEG) measures: Brain oscillatory activity during rest and evoked potentials to music stimulation are recorded for identifying biomarkers of brain state.
- Auditory Evoked Potential (AEP) measures.
- **Coma Recovery Scale - Revised (CRS-R)** (Giacino et al., 2004): The CRS-R is a unique tool which includes the current diagnostic criteria for coma, vegetative state, and the MCS, and allows the patient to be assigned to the most appropriate diagnostic category.
- **Disability Rating Scale** (Rappaport, 1982): it evaluates the functional changes in rehabilitation setting. The DR Scale consists of 8 items divided into 4 categories: Arousal and awareness; cognitive ability to handle self-care functions; physical dependence upon others; psychosocial adaptability for work, housework, or school.
- **Rancho Levels of Cognitive Functioning** (Hagen, 1979)**:** to assess cognitive functioning in post-coma patients, classifying them in one of eight level: Use of the scale generates a classification of the patient in one of eight levels:
  No response; Generalized; Localized; Confused-agitated; Confused, inappropriate, non-agitated; Confused-appropriate; Automatic-appropriate; Purposeful-appropriate.
- **Glasgow Outcome Scale – Extended**: is a global scale for functional outcome that rates patient status in eight categories according to the disability.

1. **Patients with post-stroke aphasia**

All patients with vascular or haemorrhagic post-stroke language disease consecutively admitted to Neurological Unit will enrolled in this study. Inclusion criteria are age >18 years, no auditory injury as evaluated by anamnesis, no history of neurological and psychiatric disease. Exclusion criteria are previous stroke, use of alcohol or drugs, premorbid dementia.

**Sample size determination**

We decided to test 30 patients with post-stroke aphasia (*n* = 10 per experimental paradigm) as a power analysis using G∗Power 3 suggested that this sample size will be sufficient to achieve a medium effect (f = 0.25) in a repeated-measures analysis of variance (*a* = 0.05, power (1-*b*) = 0.80) with condition as the between-subject factor (tEs combined with music stimulation vs. sham stimulation and noise vs. music stimulation and sham stimulation) and time (pre-intervention, post-intervention and post-placebo) as the within-subject factor. In the case of dropouts, we will continue recruitment and repeat the randomization procedure until at least a number of 30 participants is collected.

**Outcome measures**

Patients with aphasia will be evaluated using:

- Electroencephalography (EEG) measures: Brain oscillatory activity during rest and evoked potentials to music stimulation are recorded for identifying biomarkers of brain state.
- Auditory Evoked Potential (AEP) measures.
- **Aachener Aphasia Test (AAT)** (Luzzatti, Willmes, & De Blesere, 1996) was used to analyse language deficits and to diagnose the type of aphasia. This battery is composed by six subtests: spontaneous speech, token test, repetition, written language, naming and comprehension.
- **Italian Version of Functional Outcome Questionnaire for Aphasia (FOQ-A)** (Glueckauf et al., 2003; Ketterson et al., 2008; Spaccavento et al., 2018). This measure was administered to caregivers by the speech and language therapist to evaluate the functional communication of persons with aphasia. It consists of 32 items divided into four subscales: communicating basic needs (CBN) (seven items), making routine requests (MRR) (7 items), communicating new information (CNI) (8 items) and attention/other communication skills (AO) (10 items). The items are scored on a five-point scale, with 1 = able to successfully perform the behaviour 0% of the time; 2 = able to successfully perform the behaviour 25% of the time; 3 = able to successfully perform the behaviour 50% of the time; 4 = able to successfully perform the behaviour 75% of the time; 5 = able to successfully perform the behaviour 100% of the time. For each subscale, the items are ordered according to task difficulty.
- **Functional Assessment Measure (FAM) Cognitive Subscale** (Hall, 1992; Hall et al., 1993; Tesio & Cantagallo, 1998) This instrument includes 14 items assessing comprehension, expression, reading, writing, speech intelligibility, social interaction, emotional status, adjustment to limitation, employability, problem solving, memory, orientation, attention span and safety judgment.
- **Quality of Life Questionnaire for Aphasics (QLQA)** (Spaccavento et al., 2014) is a measure of quality of life (QOL) for persons with aphasia focusing on the ability to perform basic functions and to communicate health difficulties (e.g., to draw attention if necessary to the use of pharmacological therapy), the psychological changes due to the disability and the ability to socialize and linguistic and contextual comprehension and expression in routine daily activities. It consists of 37 items divided into three subscales: Communication Subscale (22 items) evaluating the person’s ability to express and understand real life and pragmatic situations; Psychological Condition Subscale (6 items) measuring the impact of language disorder on emotional status; Autonomy Subscale (9 items) assessing the independence of participants in performing activities of daily life. This scale was administered to the person with aphasia together with his/her caregiver.

1. **Patients with post-stroke neglect**

All patients with vascular or haemorrhagic post-stroke left visual neglect consecutively admitted to Neurological Unit will be enrolled in this study.

The inclusion or exclusion criteria are the same of patients with aphasia group.

**Sample size determination**

We decided to test 30 patients with neglect (*n* = 10 per experimental paradigm) as a power analysis using G∗Power 3 suggested that this sample size will be sufficient to achieve a medium effect (f = 0.25) in a repeated-measures analysis of variance (*a* = 0.05, power (1-*b*) = 0.80) with condition as the between-subject factor (tEs combined with music stimulation vs. sham stimulation and noise vs. music stimulation and sham stimulation) and time (pre-intervention, post-intervention and post-placebo) as the within-subject factor. In the case of dropouts, we will continue recruitment and repeat the randomization procedure until at least a number of 30 participants is collected.

**Outcome Measures**

Besides the same neurophysiological biomarkers as for the other patient groups, w e will evaluate the presence of USN by using structured tests for specific spatial domains.

For **personal neglect**, we will administer the semi-structured scale for functional evaluation of personal neglect (Zoccolotti et al., 1992). The patient will ask to demonstrate the use of 3 common objects: comb, eyeglasses, and razor for men or powder for women.

For **peripersonal neglect**, we will use the following 4 tests (Pizzamiglio et al., 1989):

Barrage test: the subject has to cross out all lines (36) on the sheet of paper.

Letter cancellation test: the subject is given an array of 6 lines, each containing 52 capital letters and has to cross out the letter H.

Sentence reading test: 6 sentences, one per sheet of paper, are presented.

Wundt-Jastrow Area Illusion Test: the stimuli are semicircular fans of identical shape and size. The subject must indicate which fan is longer.

Scores below the cutoff in 3 of the 4 tests indicate a diagnosis of peripersonal neglect.

The **extrapersonal neglect** will evaluate with the semi-structured scale for the functional evaluation of extrapersonal neglect (Zoccolotti et al., 1992). This scale includes 4 subscales: serving tea, dealing cards, picture description, and description of a room. We will use only the following 3 subscales because according to the authors, these items, unlike the picture description, evaluate extrapersonal space more specifically.

**Experimental design**

For each group, patients will be randomized into 3 groups:

1. Patients receiving tEs and subsequently music stimulation for 2 weeks, 1 week of wash out, then sham stimulation and noise (placebo) for other 2 weeks ;
2. Patients receiving only music stimulation and sham stimulation for 2 weeks, 1 week of wash out, then sham stimulation and noise (placebo) for another 2 weeks.
3. Patients receiving sham stimulation and noise (placebo) for the first 2 weeks, then 1 week wash out, and finally tEs and subsequently music stimulation for other 2 weeks;

The assessment will be carried out before enrollment, at the end of every treatment period.

**Brain stimulation**

The tEs will be applied for 20 minutes every day, 5 times a week, for 2 weeks.

Position of electrodes will be, however, adjusted according to the clinical condition of the patient.

**Music intervention**

For all patient’s groups, a specific musical anamnesis will be obtained through a structured interview with the caregiver, in order to know the patient’s lifestyle history and above all his musical tastes. The outcome of this interview will serve to tailor individual treatment protocols using familiar and self-relevant music. A similar protocol has been used in studies published in journals such as Brain and The Gerontologist and conducted by collaborators of prof. Brattico (involved in this study). Moreover, the caregivers will be instructed by the music therapist as to how to stimulate musically their relative. This music stimulation will consist of both singing familiar tunes and playing a playlist of songs that will be compiled by the music therapist based on the music anamnesis interview.

The music therapy sessions will take place for each patient as follows:

- Live music session consists in the presentation to the patients of live music, according to patient’s personal tastes and meanings for 2 days a week and musical activities selected from the neurologic music therapy (NMT) approach by Thaut (2009);
- Recorded music session consists in the presentation for 5 days a week for around 30 minutes in the morning and another 30 minutes in the afternoon of a music playlist compiled based on the musical preferences of the patients and on the music-therapeutic criteria.

**CAREGIVER DISTRESS**

To evaluate the impact of music therapy on caregiver’s burden and psychological distress will be administered the following questionnaires for all sub-groups of caregiver’s patients:

• Back Depression Inventory-II (Beck et al. 1996; Ghisi et al., 2006): to assess severity of depressive symptoms;

• State-Trait anxiety inventory (Spielberg et al., 1983; Pedrabissi et al., 1989): to assess level of anxiety;

• Questionario psicofisiologico/forma ridotta – psychophysiological inventory/reduced form: to assess psychophysiological symptoms;

• Prolonged grief disorder-12 (Prigerson et al., 1999;  Chiambretto et al., 2008): to evaluate the presence of separation distress and cognitive, behavioural or emotional symptoms at least for six months after the disease onset of a loved person;

• Family strain questionnaire (Rossi Ferrario et al., 2001): to assess perceived caregiving related problems;

• World health organization –quality of life (Harper and Power, 1998; De Girolamo et a., 2000): a questionnaire evaluating QOL in four domains: physical, psychological, social and environment relations.

**Data analysis**

The aim will be to evaluate the effects of the treatments in the various patient groups. The descriptive analysis of the data will allow us to present the characteristics of the groups. Comparison of demographic and clinical characteristics of the groups will be performed using one-way testing ANOVA for continuous variables and the Chi-square test for categorical variables. The ANOVA test for repeated measures will be used to evaluate the effects of treatments between groups on all dependent variables.

**Results**

The authors aim to highlight the efficacy of music therapy with non-invasive brain stimulation in neurological patients, assessing also the influence of this rehabilitation protocol on caregiver’s burden and psychological distress.

**References**

Aldridge D, Gustorff D, Hannich HJ. (1990) Where am I? Music therapy applied to coma patients. J R Soc Med. Jun;83(6):345-6. No abstract available.

Beck AT, Steer RA and Brown GK (1996) "Manual for the Beck Depression Inventory-II". San Antonio, TX: Psychological Corporation

Chen MC, Tsai PL, Huang YT, Lin KC. (2013) Pleasant music improves visual attention in patients with unilateral neglect after stroke. Brain Inj.27(1):75-82. doi: 10.3109/02699052.2012.722255

ChiambrettoP,MoroniL,GuarnieroG,etal..(2008)“ValidazioneitalianadelquestionarioProlongedGriefDisorder(PG‐12)”GItal MedLavErgon30:A105‐A110

De Aguiar V, Paolazzi CL, Miceli G. (2015). tDCS in post-stroke aphasia the role of stimulation parameters, behavoral treatment and patient characteristics. Cortex, 63, 296-316;

De Girolamo G, Rucci P, Scocco P, et al. (2000). Quality of life assessment: validation of the Italian version of the WHOQOL-Brief. Epidemiology and Psychiatric Sciences 9 (1), 45-55;

Dimitri D, De Filippis D, Galetto V, Zettin M. Evaluation of the effectiveness of transcranial direct current stimulation (tDCS) and psychosensory stimulation through DOCS scale in a minimally conscious subject. Neurocase. 2017 Apr;23(2):96-104. doi: 10.1080/13554794.2017.1305112. Epub 2017 Mar 27.

Ghisi M, Flebus GB, Montano A, Sanavio E, Sica C (2006). Manuale BDI-II. Beck Depression Inventory – II. Organizzazioni Speciali, Firenze.

Giacino JT1, Kalmar K, Whyte J. The JFK Coma Recovery Scale-Revised: measurement characteristics and diagnostic utility. Arch Phys Med Rehabil. 2004 Dec;85(12):2020-9.

Giacino J, Fins JJ, Machado A, Schiff ND. Central thalamic deep brain stimulation to promote recovery from chronic posttraumatic minimally conscious state: challenges and opportunities.

Glueckauf RL, Blonder LX, Ecklund-Johnson E, et al. (2003) Functional outcome questionnaire for aphasia: overview and preliminary psychometric evaluation. NeuroRehabilitation.18:281–290.

Neuromodulation. 2012 Jul;15(4):339-49. doi: 10.1111/j.1525-1403.2012.00458.x. Epub 2012 May 24. Review.

Hagen, C. , Malkmus, D. , & Durham, P. (1979). Levels of cognitive functioning. In Rehabilitation of the head-injured adult: Comprehensive physical management (pp. 87-89). Downey, CA: Professional Staff Association of Rancho Los Amigos Hospital, Inc.

Hall KM. (1992) Functional assessment measure. General rehabilitation and traumatic brain injury program evaluation. San Jose (CA): Santa Clara Valley Medical Center.

Hall KM, Hamilton BB, Gordon WA, et al. (1993) Characteristics and comparisons of functional assessment indices: Disability Rating Scale, Functional Independence Measure and Functional Assessment Measure. J Head Trauma Rehabil.;8:60–74.

Harper A and Power M (1998) Development of the World Health Organization WHOQOL-BREF Quality of Life Assessment. Psychological Medicine, 28, 551–558.

Kang K, Thaut MH. (2019). Musical Neglect Training for Chronic Persistent Unilateral Visual Neglect Post-stroke. Front Neurol.; May 8;10:474. doi: 10.3389/fneur.2019.00474. eCollection 2019

Kater K (1989). Sensory stimulation in head-injured patients. Nurs Times. Aug 9-15;85(32):61. No abstract available.

Ketterson TU, Blonder LX, Donovan NJ, et al. (2008). Reliability and validity of the functional outcome questionnaire for aphasia (FOQ-A). Rehabil Psychol. 53:215–223.

Leonardi S, Cacciola A, De Luca R, Aragona B, Andronaco V, Milardi D, Bramanti P, Calabrò RS.(2018) The role of music therapy in rehabilitation: improving aphasia and beyond. Int J Neurosci. 2018 Jan;128(1):90-99. doi: 10.1080/00207454.2017.1353981. Epub 2017 Aug 8. Review

Luzzatti C, Willmes K, De Blesere R. L’Aachener Aphasie Test (AAT), Versione Italiana. Manuale e Dati Normativi. 2nd ed. Florence: Organizzazione Speciali; 1996.

Lombardi F, Gatta G, Sacco S, Muratori A, Carolei A. (2007). The italian versione of the Coma Recovery Scale- Revised (CRS-R). Funct Neurol, 22(1), 47-61;

Magee WL, Clark I, Tamplin J, Bradt L. (2017) Music interventions for acquired brain injury. Cochrane Database Syst Rev, 1 (1), CD006787;

Magee WL, O’Kelly J. (2015). Music therapy with disorders of consciousness: current evidence and emergent evidence-based practice. Ann NY Acad Sci, 1337, 256-62;

O'Kelly J, James L2, Palaniappan R3, Taborin J4, Fachner J5, Magee WL6. et al. (2013) Neurophysiological and behavioral responses to music therapy in vegetative and minimally conscious States Front Hum Neurosci. Dec 25;7:884. doi: 10.3389/fnhum.2013.00884. eCollection 2013;

Pedrabissi, L., Santiniello, M., (1989). Manuale dell’adattamento italiano dello STAI forma Y. Firenze: Organizzazioni Speciali;

Pizzamiglio L, Judica A, Razzano C, Zoccolotti P. (1989). Toward a comprehensive diagnosis of visual-spatial disorders in unilateral brain-damaged patients. Psyhol Assess, 5:199-218;

PrigersonHG,ShearMK,JacobsSC,etal.(1999)“Consensuscriteriafortraumaticgrief:Apreliminaryempiricaltest”TheBritish lfhi63 J ournalofPsychiatry1:67‐73

Rappaport M, Hall KM, Hopkins K, Belleza T, Cope DN. (1982) Disability rating scale for severe head trauma patients: coma to community. Archives of Physical Medicine and Rehabilitation, 63: 118‐123

Spielberger, C. D., Gorsuch, R. L., Lushene, R., Vagg, P ., & Jacobs, G. A. (1983). Manual for the state-trait anxiety inventory STAI (Form Y): self-evaluation questionnaire. Palo Alto, CA: Consulting Psychologists Press;

Rossi Ferrario S, Baiardi P, Zotti AM. Assessment delle problematiche caregiving-correlate: Il Family Strain Ques-tionnaire. G Ital Med Lav Erg 2001; 23(1): 25–29;

Salazar APS, Vaz PG, Marchese RR, Stein C, Pinto C, Pagnussat AS. (2018). Noninvasive brain stimulation improves hemispatial neglect after stroke: a systematic review and meta-analysis. Arch Phys Med Rehabil, 99(2), 355-366.e1.

Särkämö T1, Tervaniemi M, Laitinen S, et al. (2008). Music listening enhances cognitive recovery and mood after middle cerebral artery stroke. Brain. Mar;131(Pt 3):866-76. doi: 10.1093/brain/awn013;

Sihvonen AJ, Leo V, Särkämö T, Soinila S.(2014) [Effectiveness of music in brain rehabilitation. A systematic review]. Duodecim. 2014;130(18):1852-60. Review;

Spaccavento S, Craca A, Del Prete M, et al. (2014). Quality of life measurement and outcome in aphasia. Neuropsychiatr Dis Treat.10:27–37.

Spaccavento S, Cafforio E, Cellamare F, Colucci A, Di Palma A, Falcone R, Craca A, Loverre A, Nardulli R, Glueckauf RL. (2018) Italian adaptation of the functional outcome questionnaire - aphasia: initial psychometric evaluation. Disabil Rehabil. 2018 Dec;40(24):2925-2930. doi: 10.1080/09638288.2017.1362042.

Tesio L, Cantagallo A. (1998). The functional assessment measure (FAM) in closed traumatic brain injury outpatients: a Raschbased psychometric study. J Outcome Meas.;2:79–96.

Thaut, M. & Hoemberg, V. (2014). Handbook of Neurologic Music Therapy. Oxford University Press, New York.

Tolle P1, Reimer M. (2003) Do we need stimulation programs as a part of nursing care for patients in "persistent vegetative state"? A conceptual analysis. Axone. Dec;25(2):20-6.

Wilson, J.T. Linday, Pettigrew, Laura E.L., & Teasdale, Graham M., Structured Interviews for the Glasgow Outcome Scale and the Extended Glasgow Outcome Scale: Guidelines for their use, Journal of Neurotrauma, vol. 15, no. 8, 1998, 573-585

Zoccolotti,P.,Antonucci,G.,&Judica,A.(1992).Psychometric characteristics of two semi-structured scales for the functional evaluation of hemi-inattention in extrapersonal and personal space. Neuropsychological Rehabilitation, 2, 179–191. doi:10.1080/09602019208401407
